# Seasonal dynamics of bacterial and archaeal methanogenic communities in flooded rice fields and effect of drainage

**DOI:** 10.3389/fmicb.2014.00752

**Published:** 2015-01-08

**Authors:** Björn Breidenbach, Ralf Conrad

**Affiliations:** Department of Biogeochemistry, Max Planck Institute for Terrestrial MicrobiologyMarburg, Germany

**Keywords:** methanogenic microbial community, rice field, pyrosequencing, 16S rRNA, season, crop rotation

## Abstract

We studied the resident (16S rDNA) and the active (16S rRNA) members of soil archaeal and bacterial communities during rice plant development by sampling three growth stages (vegetative, reproductive and maturity) under field conditions. Additionally, the microbial community was investigated in two non-flooded fields (unplanted, cultivated with upland maize) in order to monitor the reaction of the microbial communities to non-flooded, dry conditions. The abundance of Bacteria and Archaea was monitored by quantitative PCR showing an increase in 16S rDNA during reproductive stage and stable 16S rRNA copies throughout the growth season. Community profiling by T-RFLP indicated a relatively stable composition during rice plant growth whereas pyrosequencing revealed minor changes in relative abundance of a few bacterial groups. Comparison of the two non-flooded fields with flooded rice fields showed that the community composition of the Bacteria was slightly different, while that of the Archaea was almost the same. Only the relative abundance of *Methanosarcinaceae* and Soil Crenarchaeotic Group increased in non-flooded vs. flooded soil. The abundance of bacterial and archaeal 16S rDNA copies was highest in flooded rice fields, followed by non-flooded maize and unplanted fields. However, the abundance of ribosomal RNA (active microbes) was similar indicating maintenance of a high level of ribosomal RNA under the non-flooded conditions, which were unfavorable for anaerobic bacteria and methanogenic archaea. This maintenance possibly serves as preparedness for activity when conditions improve. In summary, the analyses showed that the bacterial and archaeal communities inhabiting Philippine rice field soil were relatively stable over the season but reacted upon change in field management.

## Introduction

Methane (CH_4_) is the second most important greenhouse gas after carbon dioxide (CO_2_) and has a 25 times larger global warming potential than CO_2_ (Forster et al., 2007). The global budget of atmospheric CH_4_ is on the order of 500–600 Tg per year (Forster et al., 2007) and rice fields contribute in the range of 25–300 Tg CH_4_ per year (Chen and Prinn, 2005; Bridgham et al., 2013). Rice production will probably increase, in order to feed an increasing human population (Van Nguyen and Ferrero, 2006) so that CH_4_ emission from rice fields may also increase in future. In rice fields CH_4_ is produced as end product of the anaerobic degradation of organic matter by a complex microbial community consisting of hydrolytic and fermentative bacteria, and methanogenic archaea (Zinder, 1993; Conrad, 2007). Flooded rice fields have been used as a model system for studying the functioning of anoxic methanogenic microbial communities (Conrad, 2007, 2009).

In rice fields methane is produced by two major physiological guilds, the acetotrophic and the hydrogenotrophic methanogens. Acetotrophic methanogens dismutate acetate to CH_4_ and CO_2_, while the hydrogenotrophic methanogens reduce CO_2_ with H_2_ to CH_4_ (Conrad, 2007). Molecular characterization of 16S rDNA showed a worldwide distribution of methanogens in rice fields (China, Italy, Japan and Philippines) including *Methanosarcinaceae*, *Methanosaetaceae*, *Methanobacteriales*, *Methanomicrobiales*, and *Methanocellales* (Großkopf et al., 1998; Ramakrishnan et al., 2001; Wu et al., 2006). The composition of the soil archaeal community changes if temperature is increased (Peng et al., 2008; Conrad et al., 2009) or the rice field soil is treated with organic matter such as rice straw (Conrad and Klose, 2006; Peng et al., 2008). Under field conditions, however, the archaeal communities were usually found to be rather stable even after short term drainage or extended periods of managing rice fields as upland fields (Krüger et al., 2005; Watanabe et al., 2006; Fernandez Scavino et al., 2013). In a recent study of a Korean rice field, numbers of archaea and methanogens changed by less than a factor of two throughout a cropping season (Lee et al., 2014).

In contrast to the archaeal community it has been shown that the bacterial community in rice field soil changes with time after flooding (Noll et al., 2005; Rui et al., 2009). Bacterial communities in irrigated rice fields are described as complex (Asakawa and Kimura, 2008) and differ between oxic and anoxic zones (Shrestha et al., 2007). Additionally, temporal and spatial changes in the composition of the bacterial communities with changing soil conditions were observed (Noll et al., 2005; Shrestha et al., 2009). Variations in relative abundance of dominant phyla under alfalfa-rice crop rotation system were revealed (Lopes et al., 2014) whereas pasture-rice crop rotation showed a rather stable bacterial community composition (Fernandez Scavino et al., 2013).

Moreover, archaeal and bacterial communities in the rhizosphere can be shaped by the plant species (e.g., Grayston et al., 1998; Smalla et al., 2001; Conrad et al., 2008). Several other studies demonstrated that plant type had an effect on soil microbial community structure (Marschner et al., 2001; Smalla et al., 2001; Costa et al., 2006). In addition to plant residues and soil organic matter, rhizodeposits are the major substrate input into soil (Kimura et al., 2004). Rhizodeposits are plant-derived carbon-containing compounds, which are actively secreted via the plant roots or originate from sloughed-off root cells (reviewed by Dennis et al., 2010). Rhizodeposition takes place at the zone around the plant root called rhizosphere which was shown to harbor a specific microbial community (Kowalchuk et al., 2010). Rhizodeposition depends on environmental factors, plant species, type and cultivar as well as plant age (Aulakh et al., 2001; Uren, 2007). The microbial community in the rhizosphere may be influenced by these variations in rhizodeposition.

Therefore, we hypothesized that the microbial community in rice field soil will be influenced by rice plant growth stage. Since a comprehensive seasonal record of resident and active microorganisms was lacking, we investigated the archaeal and bacterial community in the soil under field conditions by sampling three distinct plant growth stages. Additionally, the microbial community was investigated in two fields that were not flooded and were either unplanted or cultivated with upland maize in order to monitor the reaction of the rice specific microbial community to non-flooded conditions and to the presence or absence of maize. The microbial composition and abundance was assessed by fingerprinting with terminal-restriction fragment length polymorphism (T-RFLP) and quantitative PCR (qPCR) targeting the archaeal and bacterial ribosomal 16S rRNA and 16S rDNA. In order to identify changes in the lower taxonomic groups, archaeal and bacterial 16S rRNA was targeted by 454 pyrosequencing. Interestingly, we observed rather stable archaeal and bacterial communities in the soil during rice plant growth but detected more pronounced differences between flooded and non-flooded fields.

## Material and methods

### Sampling site and sample processing

The sampling site was located at the International Rice Research Institute (IRRI) in Los Banos, Philippines. Detailed site description can be found in Heinz et al. (2013). Briefly, we studied fields cultivated with irrigated rice throughout one cropping season at the vegetative (February 2012), reproductive (March 2012) and maturity (May 2012) growth phase of the rice plants (variety: NSIC Rc222). Additionally rice fields, which had been drained and were now managed as upland fields cultivating upland maize (variety: Pioneer P3482YR) were sampled after plowing and before maize seeding and fertilization as unplanted and drained rice field (unplanted) and during the reproductive growth phase of maize (maize). The study site was cropped with paddy rice in both wet and dry season over two decades (Weller et al., 2014). This season (dry 2012) was the first season in which the fields were managed as upland maize fields. Fields were operated in triplicates and managed with conventional N-fertilization (rice: seeding 30 kg N/ha, 30 kg P_2_O_5_/ha, 30 kg K_2_O/ha; at 28 and 55 days after seeding (DAS) 50 kg N/ha; maize: seeding 30 kg N/ha, 50 kg P_2_O_5_/ha, 30 kg K_2_O/ha; at 27–29 and 47-50 DAS 50 kg N/ha). In each of these fields we randomly selected three sampling plots of one square meter and sampled one soil core (5 cm diameter) from each plot. Soil cores were always taken from the vicinity of a plant (ca. 10 cm). The soil contained numerous fine roots and thus was most probably influenced by the plant roots. However, no attempts were made to separate a specific rhizospheric soil compartment. Subsequently, soil samples of 5 g were taken from the middle of the core (ca. 10 cm depth), added to 10 mL RNAlater© solution (Life Technologies, Darmstadt, Germany), kept on ice and later stored at −20°C to ensure RNA stability. For further analysis (determination of soil variables), additional samples of 50 g were taken from the same soil core and stored at −20°C.

### Determination of soil variables

For determination of soil water content small amounts of soil (1–5 g) were dried at 65°C for 3 days. The gravimetric water contents of the samples from fields cultivated with rice or maize and unplanted fields were 42.8 ± 3.5, 34.3 ± 1.2, and 36.0 ± 2.2%, respectively. The pH of the soil was analyzed following the DIN ISO 10390 protocol. Briefly, 3 g of soil was mixed with 0.01 M CaCl_2_ in a ratio of 1:2.5 and incubated rotating at 25°C for 10 min. Subsequently, the samples were incubated at 25°C for 60 min without agitation and then, after shaking the sample, the pH was measured using a pH meter (pH530 WTW, Weilheim, Germany). The pH values in rice, maize and unplanted fields were pH 6.8, 6.6, and 6.2, respectively. The soil texture was silt loam and the determination was conducted using a laser particle measuring device (LS13320, Beckmann-Coulter, Krefeld, Germany) at the geographic institute of the RTWH Aachen.

### Nucleic acid extraction

Nucleic acids were extracted following a modified version of the protocol from Bürgmann et al. (2001). Briefly, after removal of RNAlater© solution by centrifugation at 2500 × g for 2 min, 0.5 g of soil were extracted via bead-beating for 45 s at 6 m/s using a FastPrep^®^-24 (MP Biomedicals, Eschwege, Germany) in the presence of a 850 μl extraction buffer (20 ml 1 M sodium phosphate (pH 8.0), 2.5 g SDS, 10 ml 0.5 M EDTA (pH 8.0) and 2 ml 5 M NaCl). The tube was centrifuged at maximum speed for 5 min at 20°C. Then, 850 μl of phenol/chloroform/isoamylalcohol (25:24:1; Fluka, Sigma-Aldrich, Taufkirchen, Germany) was added to the supernatant and mixed. The bead beating was repeated twice using fresh extraction buffer. After mixing, the tubes were centrifuged for 5 min at maximum speed at 20°C. Then, 800 μl of chloroform/ isoamylalcohol (24:1; Fluka, Sigma-Aldrich, Taufkirchen, Germany) was added to the supernatant. After further centrifugation, 1 ml of precipitation solution (20 g PEG 6000, 16.6 g NaCl) was added to the aqueous supernatant, mixed, and kept at room temperature for 1 h. After centrifugation for 1 h with maximum speed at 4°C the sample was resuspended in 75% ice cold ethanol (Roth, Karlsruhe, Germany) and subsequently centrifuged for 10 min at maximum speed and 4°C. The resulting pellet was air dried and resuspended in 100 μl nuclease free water (Invitrogen, Darmstadt, Germany) and stored at −80°C until analysis. The total nucleic acids in 50 μl aliquot were digested with 37.5 μl nuclease free water (Invitrogen), 2.5 μl RNase-free DNase and 10 μl buffer RDD (Qiagen, Hilden, Germany) at room temperature for 10 min. The digest was then purified using RNeasy kit (Qiagen) following the RNA Cleanup protocol in the manufacturer's instructions. Complete DNA removal was verified by failure to obtain a PCR amplification product of bacterial 16S rDNA with the purified RNA template using the conditions described below. cDNA was synthesized from purified RNA using SuperScript™ III reverse transcriptase (Invitrogen) according to the manufacturer's instructions. Random hexamer primers (50 ng/μl) were used for complete cDNA synthesis which was used for amplification of the archaeal and bacterial 16S rRNA.

### Quantitative polymerase chain reaction

The quantification of archaeal and bacterial 16S rDNA/rRNA was conducted using quantitative polymerase chain reaction (qPCR) using primer combinations Ba519f/Ba907r (Stubner, 2002) for bacterial and Ar364f (Burggraf et al., 1997) / Ar934br (Großkopf et al., 1998) for archaeal genes. The qPCR was conducted in 96-well micro titer plates (BioRad, München, Germany) using an iCycler MyiQ™ (BioRad). Each qPCR reaction contained in a total volume of 25 μl, 1 × SYBRGreen Ready Mix (Sigma), 3 mM MgCl_2_ (Sigma), 0.25–0.66 μM of each primer and 1 μM FITC (fluorescein thiocyanat; BioRad) as well as 1–2 μl target DNA respectively cDNA. Purity of the used reagents was ensured using negative controls not containing any DNA matrix. The DNA standard prepared from clones containing bacterial or archaeal 16S rDNA in a plasmid insert was applied in a dilution series containing 1 × 10^7^ to 1 × 10^1^ gene copies. The thermal profile used for amplification included 40 to 50 cycles of denaturation at 94°C for 30 s, primer annealing at 50°C (Ba519f /Ba907r) or 66°C (Ar364/Ar934br) for 20–30 s and primer extension at 72°C for 45 s. Afterwards a melting curve from 75 to 95°C (0.2°C s^−1^) was performed in order to confirm specificity of the real time PCR reaction. The data were analyzed using BioRad IQ5 2.0 Standard Edition Optical System software (Biorad).

### Terminal fragment length polymorphism (T-RFLP)

T-RFLP analysis of archaeal and bacterial 16S rRNA and rDNA was conducted based on fractionation of terminal fluorescence-labeled PCR products after use of restriction enzymes as described (Chin et al., 1999) using the primer combination Ar109f (Großkopf et al., 1998)/Ar912rt-FAM (Lueders and Friedrich, 2003) and Ba27f-FAM (Osborne et al., 2005)/Ba907r (Muyzer et al., 1995), respectively. All PCR reactions were performed in a total volume of 50 μl. For amplification each reaction contained 5 × Green GoTaq^®^ Flexi buffer (Promega, Mannheim, Germany), 200 μM deoxy-nucleoside triphosphates (dNTPs; Fermentas, St. Leon-Rot, Germany), 0.5 μM of each primer, 10 μg bovine serum albumin (BSA; Roche, Grenzach, Germany), 1 U GoTaq^®^ Flexi DNA polymerase (Promega) and 1 μl DNA matrix (in most cases diluted to a concentration of 20 ng/μl). All amplifications were carried out in a GenAmp 9700 Thermocycler (Applied Biosystems, Carlsbad CA, USA). The thermal profile used for amplification included 25 to 30 cycles of primer annealing at 52°C for 45 s, primer extension at 72°C for 90 s, and denaturation at 94°C for 45 s. PCR product purification was conducted using the GenElute™ PCR Clean-up kit (Sigma) following the manufacturer's instructions. The purified amplicons were digested by using *MspI* (cutting side: 5′-C▼CGG-3′, 37°C; Fermentas) for bacterial and *TaqI* (cutting side: 5′-T▼CGA-3′, 65°C; Fermentas) for archaeal 16S rDNA and rRNA. The fragmented DNA was purified using SigmaSpin™ Post Reaction Clean-Up columns (Sigma) following the manufacturer's instructions. T-RFLP reactions contained 0.2 μl size standard (X-rhodamine MapMarker^®^ 1000, BioVentures, Murfreesboro, USA). Separation was accomplished using capillary electrophoresis in an ABI PRISM 3130 Genetic Analyzer (Applera). Data analysis was conducted using GENESCAN Analysis software 4.0 (Applied Biosystems, Carlsbad, USA). Normalization and standardization of the T-RFLP profiles was done according the method from Dunbar et al. (2001). The relative abundance was calculated from the ratio between the height of the fluorescence signal and the total height of all signals in one sample.

### Cloning and sequencing

A clone library of archaeal 16S rDNA was created for subsequent phylogenetic classification. The pGEM^®^-T Easy Vector System (Promega) was used. The purified PCR products were ligated into the pGEM^®^-T Easy Vector according to the manufacturer's instructions. Each ligation reaction contained 5 μl 2X Rapid Ligation Buffer (Promega), 1 μl pGEM^®^-T Easy Vector (50 ng; Promega), 1 μl T4 DNA ligase (3 Weiss units/μl; Promega) and 50 ng PCR product. Sterile water was added to reach a total volume of 10 μl. The transformation of *Escherichia coli* JM109 high efficient competent cells (Promega) was carried out according to the manufacturer's instructions. Randomly chosen white colonies were sequenced using Sanger sequencing. The received raw data (electropherograms) were processed using the program Seqman II (DNAStar, Madison, USA). The phylogeny of the archaeal sequences was analyzed using the ARB software (http://www.arb-home.de/). For the archaeal 16S rRNA sequences the public available database was downloaded from SILVA homepage (http://www.arb-silva.de/) and integrated into ARB. Alignment was conducted using the Fast Aligner tool in ARB (Ludwig et al., 2004). The alignment was then manually checked and where necessary corrected. Subsequently, the aligned sequences were calculated into the archaeal 16S rRNA tree under usage of the neighbor-joining algorithm as described in detail by Wu et al. (2006). The restriction sites characteristic for the fragment length of the T-RFs were determined. The T-RFs determined from T-RFLP analyses were assigned to the corresponding clones and their phylogeny. The archaeal 16S rRNA sequences data have been submitted to the GenBank databases under accession numbers: KM463011–KM463082.

### 454 pyrosequencing

Tagged pyrosequencing of the bacterial and archaeal community was conducted using primer combinations F515/R806 (Bates et al., 2011) and Arch344F (Casamayor et al., 2002)/A934br (Großkopf et al., 1998), respectively. The forward primers were tagged with a unique 8-base pair barcode. Sequencing of the PCR products was done at the Max Planck Genome Centre in Cologne using a Roche 454 Genome Sequencer GS FLX+. Data analysis was performed using mothur software package version 1.31.2 (http://www.mothur.org/) following the standard operational procedure including sequence quality management (SOP, Schloss et al., 2009). OTU clustering and analysis was conducted using UPARSE pipeline as described by Edgar (2013). Only microbial high-quality sequences with a minimum read length of 200 bp were used. Sequences that did not match the primer sequences and were smaller than 200 bp or contained any ambiguities were excluded from further analysis. After denoising, sequences were aligned against the SILVA bacteria/archaea 16S rDNA database (Schloss et al., 2011; Pruesse et al., 2007). Sequences which were not assigned to bacteria or respectively archaea were discarded. Operational taxonomic units (OTU) were defined using a distance matrix with 3% dissimilarity (Zinger et al., 2011). Further analyses including rarefaction curves, species richness and diversity indices were conducted as described in the SOP pyrosequencing pipeline (Schloss et al., 2011). An overview of the number of sequences retrieved and the accession numbers of the submitted sequences can be found in **Tables 2, 3**.

### Statistical analysis

Statistical analyses were done in R version 2.14.1 (R Development Core Team, 2011). If necessary, normal distribution was achieved by log-transforming the data. Analysis of variance (ANOVA), PERMANOVA (ADONIS) and canonical correspondence analysis (CCA) were conducted with package vegan version 2.0.5 (Oksanen et al., 2012). All levels of significance were defined at *P* ≤ 0.05. Ternary plots were created using package vcd version 3.0.3.

## Results

### Bacterial and archaeal 16S rDNA/rRNA copy numbers

**Figure 1:**
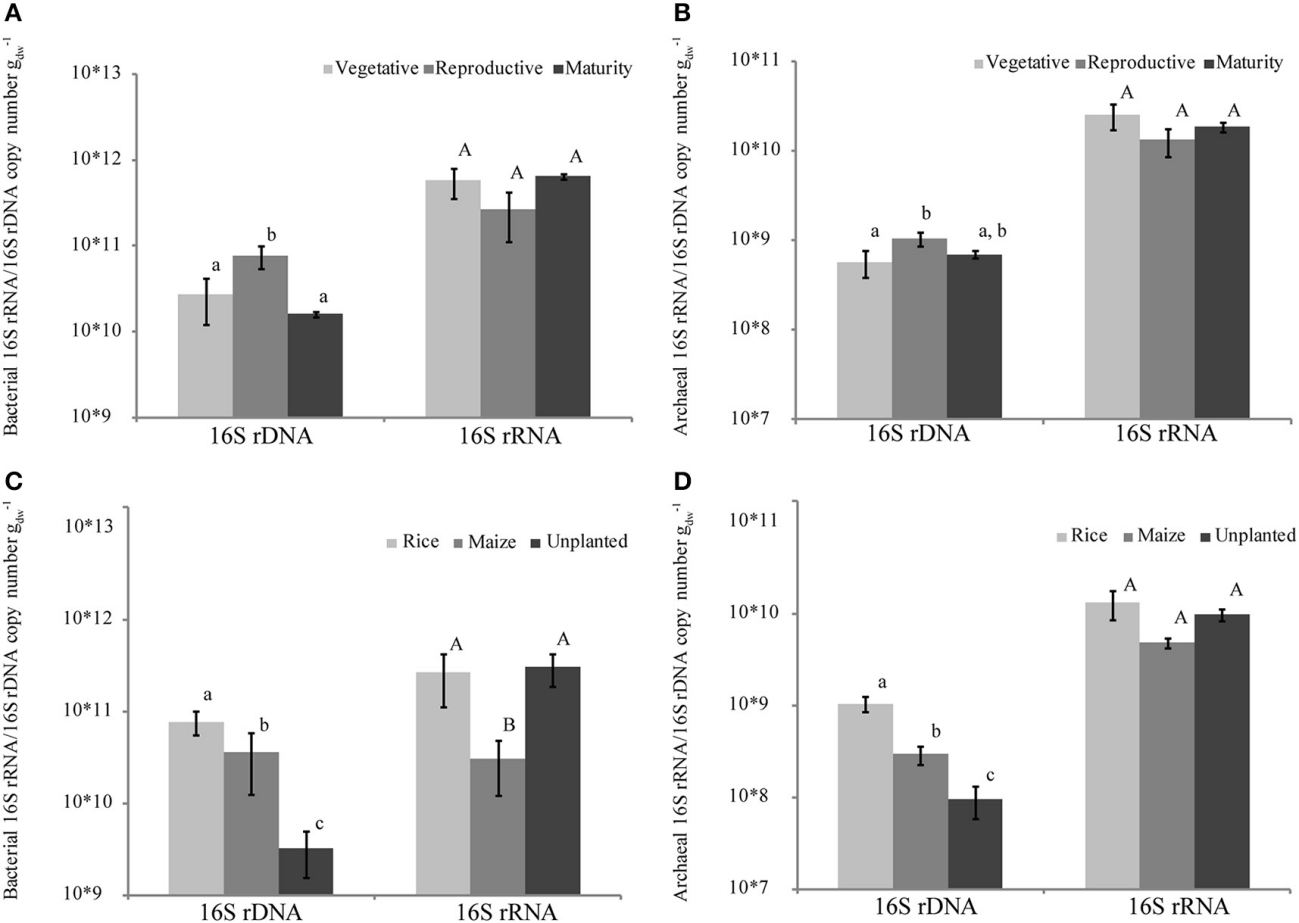
**Ribosomal 16S rDNA and rRNA copy numbers quantified using qPCR**. Abundance of bacterial 16S rDNA and rRNA **(A,C)** and archaeal 16S rDNA and rRNA **(B,D)** in rice fields at different plant growth stages **(A,B)** as well as in fields planted with rice, maize or unplanted at the reproductive growth stage **(C,D)**. Different letters indicate significant difference (mean ± *SE*, *n* = 9).

**Figure 2:**
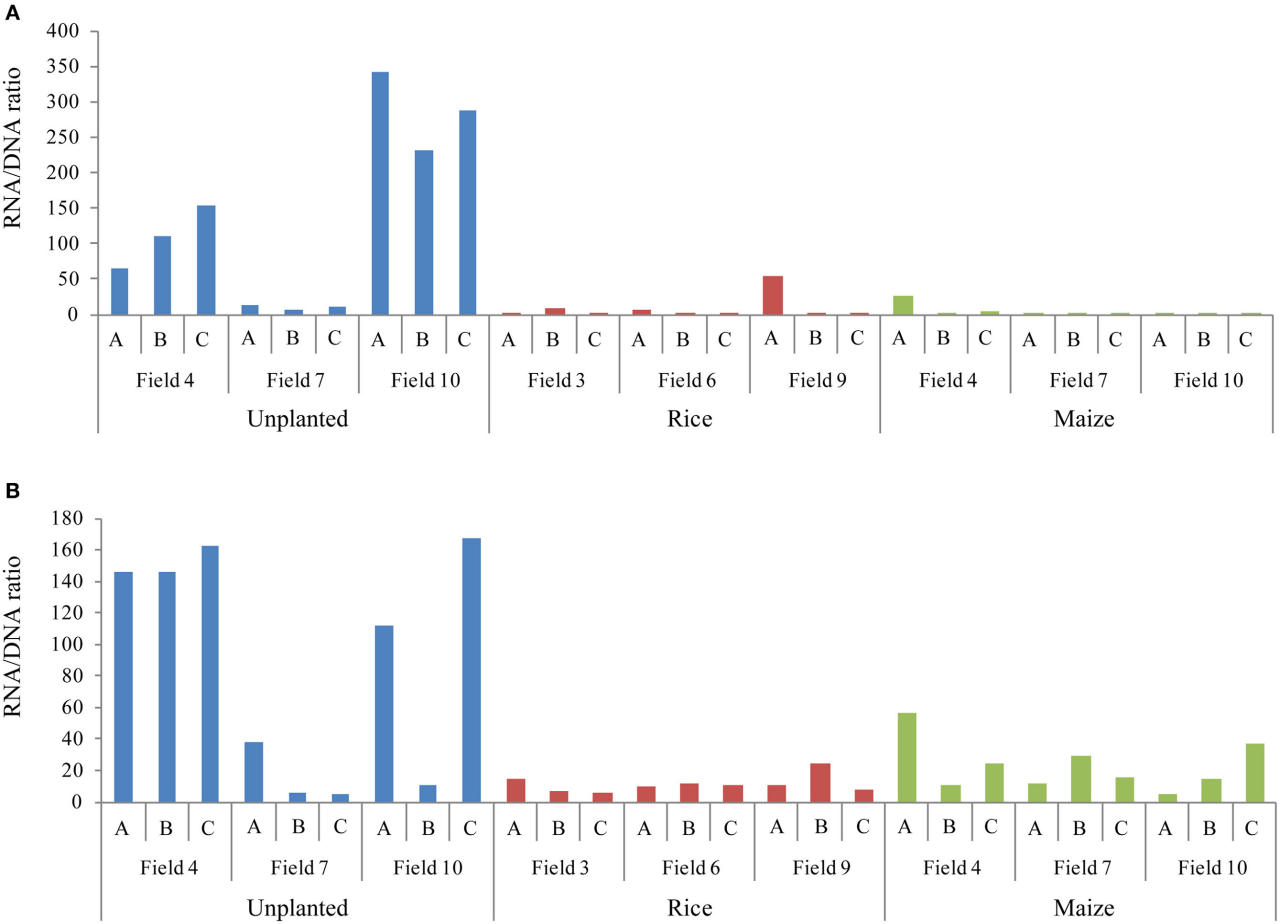
**Ratio of ribosomal 16S rRNA and rDNA copy numbers quantified using qPCR**. Bacterial **(A)** and archaeal **(B)** ratios are shown for each replicate in the unplanted, rice and maize cultivated fields.

For quantification of bacteria and archaea in rice field soil during rice plant growth we used quantitative PCR (qPCR) targeting the bacterial and archaeal 16S ribosomal RNA (16S rRNA) and their genes (16S rDNA). Copy numbers of bacterial and archaeal 16S rDNA and rRNA were quantified at three different growth stages and in differently cropped fields (Figure 1). Both bacterial and archaeal 16S rDNA copy numbers were highest during rice growth at reproductive stage, whereas the 16S rRNA copy numbers were constant during the whole season (Figures 1A,B). Comparing rice and maize cultivated soils during the reproductive growth phase, the highest copy numbers of 16S rDNA and rRNA were detected in the rice fields (Figures 1C,D). The unplanted fields contained less 16S rDNA copies than the fields cultivated with either rice or maize (Figures 1C,D). However, the numbers of archaeal and bacterial 16S rRNA copies were similar to those in the rice fields (Figures 1C,D) resulting in a high ratio of 16S rRNA/rDNA copies (Figure 2). In contrast, bacterial 16S rRNA copies were lower in the maize field than in the rice cultivated and unplanted fields (Figure 1C). Although the ratio of bacterial and archaeal rRNA/rDNA copies was significantly increased in unplanted fields in comparison to the fields cultivated with either rice or maize across all the replicates sampled, samples from replicate field 7 did not show such increase (Figure 2). The behavior of these particular replicates could not be explained by analyzing possible correlation with soil characteristics (contents of carbon, nitrogen, sulfate, nitrate, water).

### Bacterial and archaeal community analyzed by T-RFLP

**Figure 3:**
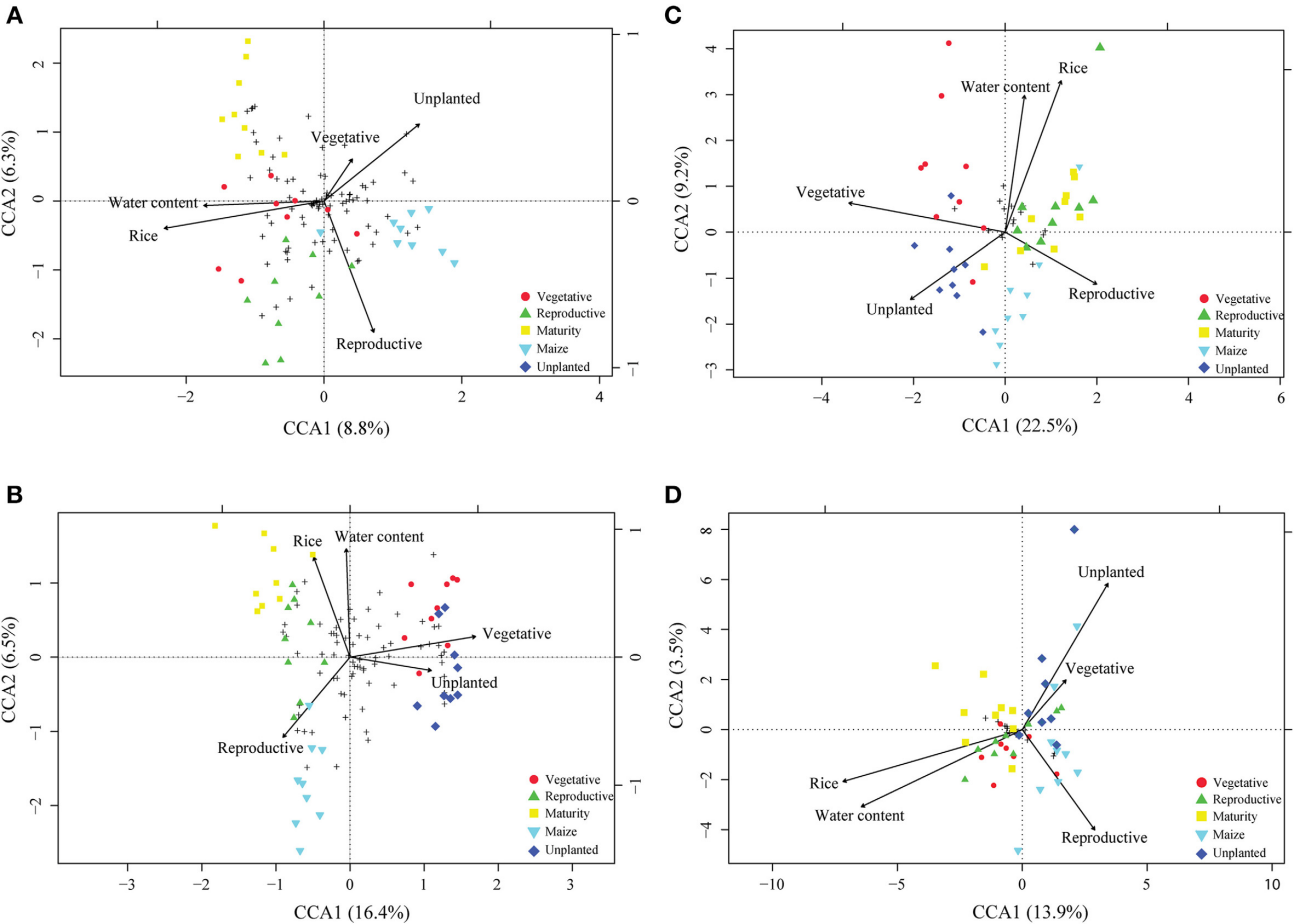
**Canonical correspondence analysis (CCA) biplot of T-RFLP based on bacterial and archaeal communities**. T-RFLP based communities of bacterial 16S rDNA and rRNA **(A,B)** as well as archaeal 16S rDNA and rRNA **(C,D)** are displayed. Arrows indicate the direction and relative importance (arrow lengths) of environmental variables associated with bacterial and archaeal community structures, respectively. Solely the environmental variables significantly influencing the model were displayed (ANOVA *p* < 0.05). Circle, triangle and square symbols, respectively, represent vegetative, reproductive and maturity growth phase of rice. Inverted triangle and diamante symbols, respectively, characterize samples originating from maize and unplanted fields while crosses represent T-RFs.

**Figure 4:**
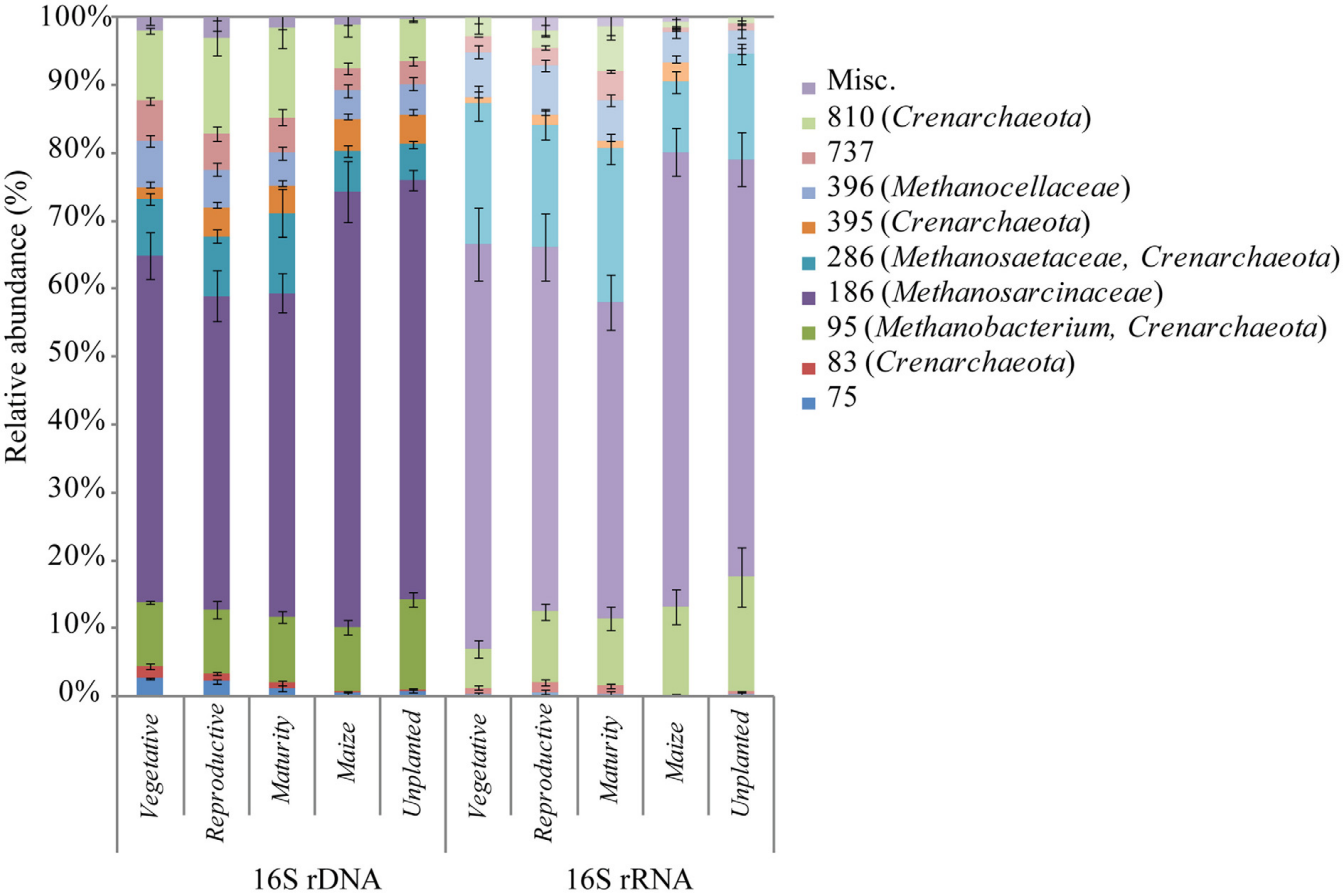
**Histograms of the relative abundance of T-RFs obtained from T-RFLP analysis of archaeal 16S rDNA and rRNA during rice plant growth**. Terminal restriction fragment sizes and affiliated clone taxonomy are given in brackets. Bars represent standard errors of *n* = 9.

For community profiling we used T-RFLP targeting the bacterial and archaeal 16S rDNA and rRNA. To identify parameters which significantly explain the variance in the microbial community, canonical correspondence analysis (CCA) was performed. Field management (rice, maize, unplanted), growth stage (vegetative, reproductive, maturity) and gravimetric water content were identified to significantly affect the microbial community explaining 6–23% of the variance (Figures 3A–D). The pure effect of each factor is shown in Supplement Table 1, with field management explaining 12–16%, growth stage 11–23% and gravimetric water content 6–12% of the variance. Although these factors were significant, the resident bacterial (Supplement Figure 1) and the archaeal (Figure 4) community composition did not change significantly during rice plant growth (ADONIS, P > 0.05). The non-flooded fields (unplanted and maize) also revealed a bacterial and archaeal community composition that was not significantly different from the rice field community (ADONIS, *P* > 0.05). Only the archaeal T-RF of 186 bp significantly increased during maize cultivation in comparison to the rice fields (Figure 4; ANOVA, *P* < 0.05). The more active bacterial and archaeal communities (16S rRNA) showed only minor variations in relative abundance of T-RFs during rice plant growth and in the unplanted and maize fields. These variations were not statistically significant (ADONIS, *P* > 0.05).

The archaeal T-RFs were assigned to different archaeal lineages by sequence analysis (Table 1). The assignment was based on a clone library of 16S rDNA containing 72 randomly selected clones retrieved from soil cultivated with rice and maize. The major T-RFs of 95, 186, 286, 396, and 810 bp were assigned as *Methanobacteriales*, *Methanosarcinaceae*, *Methanosaetaceae*, *Methanocellales*, and Miscellaneous *Crenarchaeota* respectively (Table 1). Some additional T-RFs of minor relative abundance were detected at 75, 308, 611, 671, 682, 695, 737, and 771 bp (most of them are too minor to be shown in Figure 4), which were not represented in the clone library, and therefore could not be assigned. A few clones, which were solely found in the clone library but not in the T-RFLP analysis, were assigned as Miscellaneous *Crenarchaeota* (97, 263, 333, 656, 725 bp).

**Table 1:** **Lengths of distinct terminal restriction fragments (T-RFs) of different archaeal 16S rDNA clones obtained rice and maize cultivated Philippine rice field soil and affiliation with a distinct phylogenetic lineage by 16S rDNA sequence analysis of the clones**.

**Phylogenetic affiliation**	**Terminal restriction fragment lenght (bp)**	**No. of clones rice field**	**No. of clones maize field**
Miscellaneous *Crenarchaeota*	83, 95, 191, 254, 263, 286, 379, 395, 656, 725, 795, 810	27	28
Soil Crenarchaeotic Group	191	–	1
*Crenarchaeota* Group C3	380	–	1
*Methanobacteriales*	95	1	–
*Methanosarcinaceae*	186	9	2
*Methanosaetaceae*	286	1	–
*Methanocellales*	396	1	1

### Pyrosequencing of bacterial 16S rDNA and rRNA

**Figure 5:**
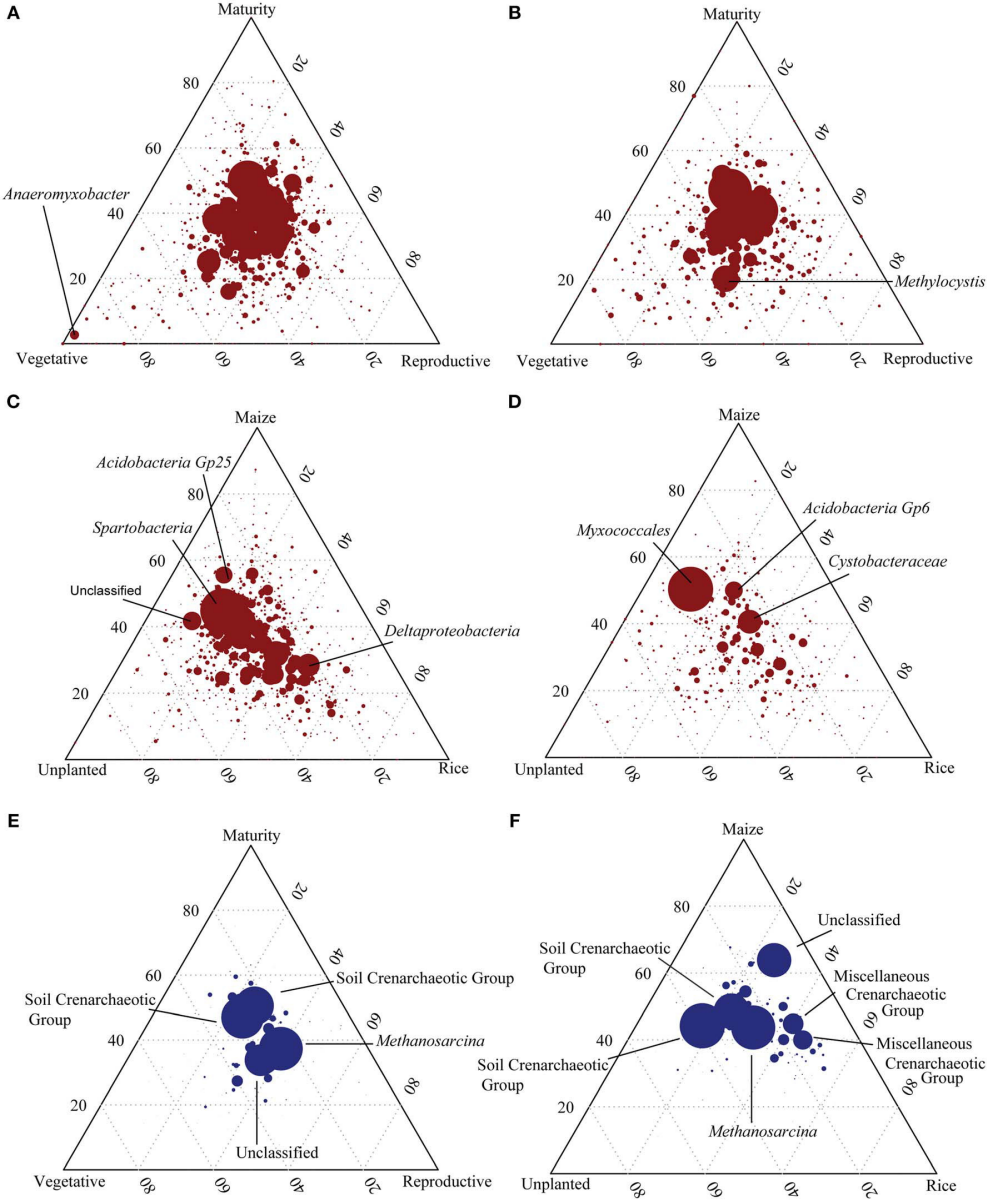
**Ternary plots showing the distribution of bacterial and archaeal 16S rDNA and rRNA based OTUs**. Axes represent rice plant growth stages (vegetative, reproductive and maturity) **(A,B,E)** as well as unplanted and maize cultivated fields in comparison to rice cultivated fields **(C,D,F)** and the percentage of reads associated with each sample for each OTU. Bacterial 16S rDNA (red, **A,C)** and 16S rRNA **(B,D)** as well as archaeal 16S rDNA (blue, **E,F**) are displayed. Each circle represents an individual OTU while its size indicates number of reads associated. The position of each OTU is determined by the contribution of the sample type to the total count (*n* = 3).

Pyrosequencing targeting the bacterial 16S rDNA and rRNA was conducted in order to indentify the resident and the active bacterial phylotypes in the Philippine rice field soil and to monitor the influence of plant growth stage on the bacterial community composition. Therefore, triplicate samples were sequenced for each growth stage resulting in 3468 to 11,311 high quality sequences of rDNA as well as 2062 to 10,275 sequences of rRNA (Table 2). For bacterial rDNA, the most dominant phylum was *Proteobacteria* (23–32%) followed by *Acidobacteria* (16–20%). Other important bacterial phyla were *Chloroflexi* (8–10%), *Verrucomicrobia* (5–6%), *Firmicutes* (4–5%), *Actinobacteria* (2–3%), *Planctomycetes* (2%), and *Cyanobacteria* (1–3%) (Supplement Figures 2A–E). The bacterial community composition did not change dramatically during the rice growing season (Supplement Figures 2A–C). Comparison of the dominant OTUs retrieved at different rice plant growth stages showed a uniform distribution over the season (Figure 5A). Only OTUs with a minor relative abundance were distinct for a particular growth stage, e.g., OTU 396 identified as *Anaeromyxobacter*, which was only found at the vegetative growth stage (Figure 5A). Comparison of unplanted, maize and rice cultivated fields showed more pronounced differences among bacterial OTUs. The OTUs number 1 (*Spartobacteria*), 4 (Unclassified) and 7 (*Acidobacteria Gp25*) were more abundant in the unplanted and in the upland maize fields than in the rice fields, while OTU number 9 (*Deltaproteobacteria*) was relatively more abundant in the rice fields (Figure 5C). Additionally, unplanted fields as well as fields cultivated with upland maize showed significantly less *Proteobacteria* in comparison to rice fields (Supplement Figures 2A,D,E). The lower relative abundance of the *Proteobacteria* was due to the lower abundance of *Geobacteraceae*. Otherwise, however, the bacterial community composition was not affected by the rice growth stage or the type of crop (ADONIS, *P* > 0.005).

**Table 2:** **Number of bacterial sequences before and after quality management, barcode, number of OTUs, coverage, Chao1 and inverted Simpson index of the environmental samples analyzed by 454-pyrosquencing**.

**Gene**	**Name**	**Growth Stage**	**Plant**	**Barcode**	**Accession**	**Raw seqs**	No. seqs^*^	**No. OTU**	**Good's coverage**	**Chao1**	**1/Simpson**
*16S rDNA*	RWVF3	Vegetative	Rice	ACGTAC	SRS715481	9579	6472	2089	0.83	3794	585
	RWVF6	Vegetative	Rice	ACTGCA	SRS715482	10001	6551	2092	0.83	3694	483
	RWVF9	Vegetative	Rice	AGAGTC	SRS715483	7818	4985	1749	0.81	3110	575
	RWRF3	Reproductive	Rice	ATCGAT	SRS715487	10831	7284	2301	0.84	4076	606
	RWRF6	Reproductive	Rice	ATGCTA	SRS715488	13418	7135	2055	0.85	3636	503
	RWRF9	Reproductive	Rice	CACAGT	SRS715489	4950	3111	1328	0.74	2739	532
	RWMF3	Maturity	Rice	CGCGCG	SRS715493	8464	5920	1613	0.86	2878	338
	RWMF6	Maturity	Rice	CGTATA	SRS715494	4079	2815	1255	0.73	2506	546
	RWMF9	Maturity	Rice	GACTAG	SRS715495	6081	3225	1339	0.75	2836	566
	MMRF4	Repoductive	Maize	CAGTCA	SRS715490	6108	3290	1269	0.78	2575	454
	MMRF7	Reproductive	Maize	CATGAC	SRS715491	5609	3112	1244	0.77	2321	473
	MMRF10	Reproductive	Maize	CGATAT	SRS715492	5449	3689	1331	0.80	2494	345
	MMVF4	–	Unplanted	AGCTGA	SRS715484	11301	7849	2233	0.86	3850	521
	MMVF7	–	Unplanted	AGTCAG	SRS715485	5719	3102	1251	0.77	2419	520
	MMVF10	–	Unplanted	ATATCG	SRS715486	7679	4264	1470	0.81	2572	442
*16S rRNA*	RWVF3	Vegetative	Rice	ACGTAC	SRS715481	12186	10275	2332	0.91	3501	511
	RWVF6	Vegetative	Rice	ACTGCA	SRS715482	8936	6732	1952	0.86	3141	533
	RWVF9	Vegetative	Rice	AGAGTC	SRS715483	9028	6831	1939	0.86	3302	464
	RWRF3	Reproductive	Rice	ATCGAT	SRS715487	10353	7672	2166	0.86	3606	586
	RWRF6	Reproductive	Rice	ATGCTA	SRS715488	12335	7107	1990	0.87	3230	459
	RWRF9	Reproductive	Rice	CACAGT	SRS715489	6965	4713	1560	0.84	2505	510
	RWMF3	Maturity	Rice	CGCGCG	SRS715493	5812	4344	1374	0.84	2371	304
	RWMF6	Maturity	Rice	CGTATA	SRS715494	3070	2062	980	0.71	1983	467
	RWMF9	Maturity	Rice	GACTAG	SRS715495	5440	3129	1249	0.78	2274	387
	MMRF4	Repoductive	Maize	CAGTCA	SRS715490	10351	5974	1675	0.86	2784	223
	MMRF7	Reproductive	Maize	CATGAC	SRS715491	7890	4481	1517	0.83	2520	229
	MMRF10	Reproductive	Maize	CGATAT	SRS715492	6226	4438	1422	0.85	2225	304
	MMVF4	–	Unplanted	AGCTGA	SRS715484	7452	6039	1790	0.86	2918	455
	MMVF7	–	Unplanted	AGTCAG	SRS715485	9622	6399	1781	0.87	2890	296
	MMVF10	–	Unplanted	ATATCG	SRS715486	10313	6230	1755	0.87	2859	466

Raw data were deposited under the study accession numbers SRP047272 for bacterial sequences in the NCBI Sequence Read Archive (SRA). Sample from one field (A–C) was randomly chosen and analyzed as representative of the field.

* number of sequences after quality analysis.

Partial 16S rRNA primers: Bacteria: F515 (5′-GTGCCAGCNGCCGCGGTAA), R806 (5′-GGACTCVSGGGTATCTAAT).

Adaptor primes: forward (5′-GATGGCCATTACGGCC), reverse (5′-GGTGGCCGAGGCGGCC).

The sequences of ribosomal RNA presumably represent the more active bacterial community. This community was composed of the same phyla as the ribosomal gene-based community, but exhibited a different composition (Supplement Figures 2F–J). The most dominant phyla within the bacterial rRNA community were *Proteobacteria* (36–40%) followed by *Acidobacteria* (14–18%). Other important bacterial phyla were *Chloroflexi* (3–4%), *Verrucomicrobia* (4–6%), *Firmicutes* (3–4%), *Actinobacteria* (2–4%), *Planctomycetes* (5–6%), and *Cyanobacteria* (3–9%) (Supplement Figures 2F–J). At the phylum level the bacterial community was not significantly different between the different plant growth stages (ADONIS, *P* > 0.005). Nevertheless, specific bacterial groups changed in abundance during rice plant growth. Only OTUs with relatively low abundance were characteristic for individual growth stages, whereas the dominant OTUs were equally distributed and observed at all three growth stages. Only OTU number 4 (*Methylocystis*) was more prominent at the vegetative and reproductive than at the maturity stage (Figure 5B). Additionally, *Verrucomicrobia* and *Bacteroidia* increased from vegetative to reproductive growth stage, *Cyanobacteria* decreased (data not shown). Comparison of the ribosomal OTUs in unplanted, maize and rice-cultivated soils showed more pronounced preferences (Figure 5D). The most dominant OTU number 1 (*Myxococcales*) was preferentially associated with the non-flooded fields (unplanted, maize) while OTU number 3 (*Acidobacteria Gp6*) and 2 (*Cystobacteraceae*) were found in all field types. Additionally unplanted fields showed higher *Bacteroidetes* and *Sphingobacteria* than the rice fields (ANOVA, *p* < 0.05). Less *Verrucomicrobia* were detected in the unplanted fields in comparison with rice and maize fields.

**Figure 6:**
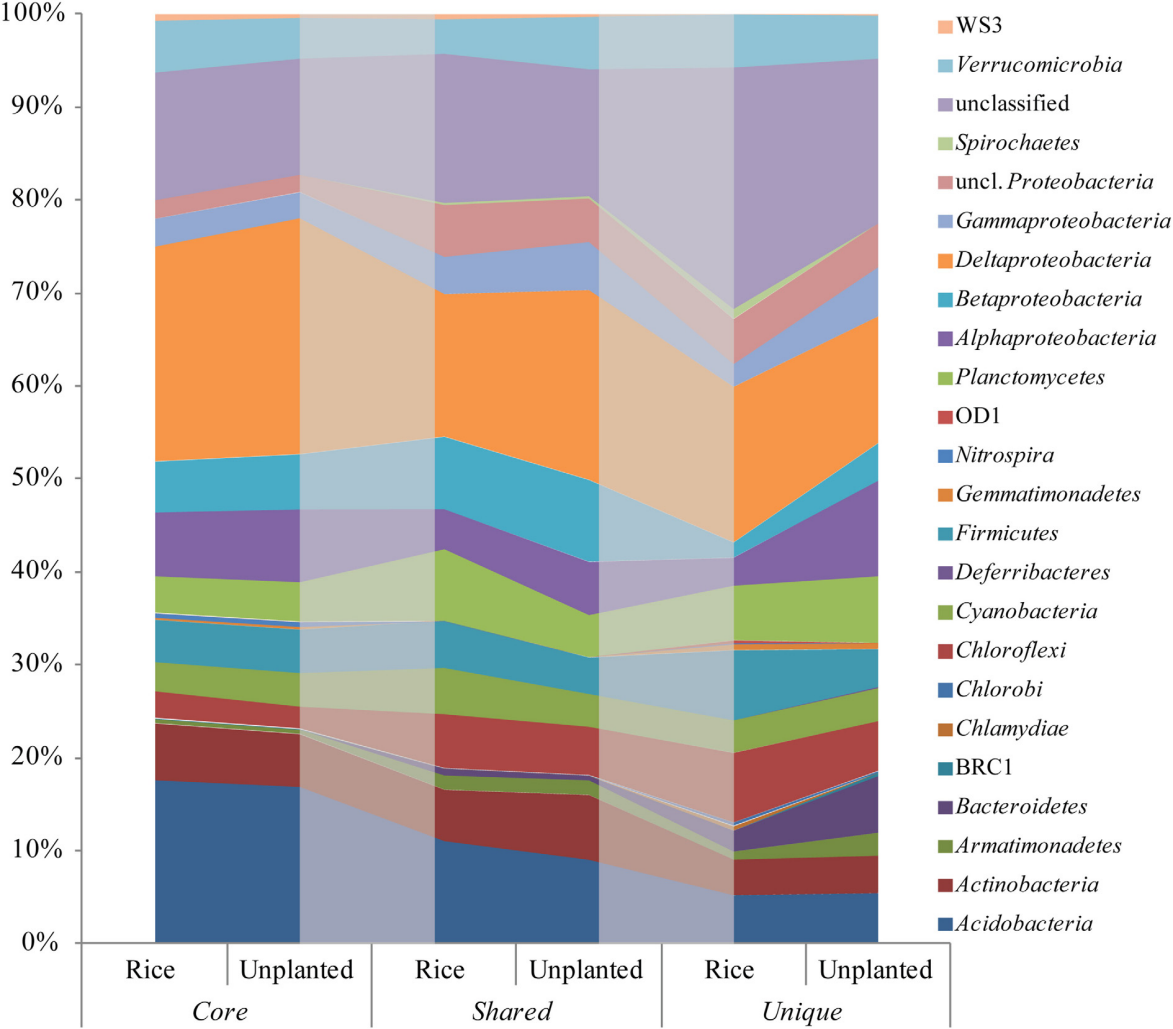
**OTU based relative sequence abundance of bacterial phyla based on 16S rRNA in the rice and unplanted fields**. OTUs detected in all fields (core), only in unplanted and rice cultivated fields (shared) and OTUs only detected in rice or unplanted fields (unique) were presented. The shaded areas serve visualization and have no special meaning.

The analysis of presence and absence of individual OTUs based on rRNA revealed that the OTUs detected in all fields (core OTUs) constituted 70, 74, and 71% of the relative abundance in rice, maize and unplanted fields, respectively. Direct comparison of the rice and the unplanted fields showed similar distribution of bacterial lineages in core, shared and unique OTUs (Figure 6). The relative abundance of core OTUs assigned as *Deltaproteobacteria* and unique OTUs assigned as *Armantimonadetes*, *Bacteroidetes*, *Alphaproteobacteria*, and *Gammaproteobacteria* was increased in the unplanted fields (Figure 6).

### Pyrosequencing of archaeal 16S rDNA and rRNA

Analogously to the bacterial community, the archaeal community was analyzed using pyrosequencing targeting archaeal 16S rDNA and rRNA. Sequencing resulted in 1326 to 9290 high quality sequences of rDNA as well as 532 to 1962 sequences of rRNA (Table 3). For archaeal rDNA, the major taxa with a relative abundance of >2% in at least one sample are shown in Supplement Figure 3A. The most dominant class was *Methanomicrobia* (40–50% relative abundance) followed by Soil Crenarchaeotic Group (20–34%), Misc. Crenarchaeotic Group (17–23%), and *Methanobacteria* (2–6%). The class *Methanomicrobia* was further subdivided into orders and families. The most dominant archaeal order was *Methanosarcinales* with the families *Methanosarcinaceae*, *Methanosaetaceae*, and the group GOM Arc I (Supplement Figure 3A). Comparison of the dominant OTUs retrieved at different rice plant growth stages showed a uniform distribution over the season (Figure 5E). Most dominant OTUs were assigned as Soil Crenarchaeotic Group and *Methanosarcina* and their distribution did not change in composition during rice plant growth (Figure 5E). Several trends, albeit not significant, are worth mentioning. Thus, the relative abundance of *Methanobacteriaceae*, *Methanosarcinaceae*, *Methanosaetaceae*, and *Methanocellaceae* was relatively high in the reproductive stage, while that of GOM Arc I was relatively low (Supplement Figure 3A). The relative abundance of the genera *Methanolinea* and Candidatus *Methanoregula* decreased from vegetative to later growth stages.

**Table 3:** **Number of archaeal sequences before and after quality management, barcode, number of OTUs, coverage, Chao1 and inverted Simpson index of the environmental samples analyzed by 454-pyrosquencing**.

**Gene**	**Name**	**Growth Stage**	**Plant**	**Barcode**	**Accession**	**Raw seqs**	No. seqs^*^	**No. OTU**	**Good's coverage**	**Chao1**	**1/Simpson**
*16S rDNA*	RWVF3	Vegetative	Rice	ACGTAC	SRS715481	4063	1956	164	0.97	208	32
	RWVF6	Vegetative	Rice	ACTGCA	SRS715482	2572	1326	140	0.96	205	24
	RWVF9	Vegetative	Rice	AGAGTC	SRS715483	4886	2828	177	0.98	293	29
	RWRF3	Reproductive	Rice	ATCGAT	SRS715487	7437	3753	231	0.98	359	30
	RWRF6	Reproductive	Rice	ATGCTA	SRS715488	5458	2410	188	0.97	264	32
	RWRF9	Reproductive	Rice	CACAGT	SRS715489	1414	929	112	0.95	168	18
	RWMF3	Maturity	Rice	CAGTCA	SRS715493	6656	3630	202	0.98	291	25
	RWMF6	Maturity	Rice	CATGAC	SRS715494	4312	2137	176	0.97	284	31
	RWMF9	Maturity	Rice	CGATAT	SRS715495	6515	3250	201	0.97	492	26
	MMRF4	Repoductive	Maize	CAGTCA	SRS715490	8727	4644	206	0.99	256	17
	MMRF7	Reproductive	Maize	CATGAC	SRS715491	7723	3378	192	0.98	251	29
	MMRF10	Reproductive	Maize	CGATAT	SRS715492	6280	2894	164	0.99	194	23
	MMVF4	–	Unplanted	AGCTGA	SRS715484	6678	3369	168	0.99	215	10
	MMVF7	–	Unplanted	AGTCAG	SRS715485	2918	1504	128	0.97	185	16
	MMVF10	–	Unplanted	ATATCG	SRS715486	3909	2049	159	0.98	208	23
*16S rRNA*	RWRF3	Reproductive	Rice	ATCGAT	SRS715487	3232	1962	169	0.97	232	36
	RWRF6	Reproductive	Rice	ATGCTA	SRS715488	2797	1727	128	0.97	239	25
	RWRF9	Reproductive	Rice	CACAGT	SRS715489	572	565	74	0.95	126	19
	MMRF4	Repoductive	Maize	CAGTCA	SRS715490	7914	804	74	0.96	115	8
	MMRF7	Reproductive	Maize	CATGAC	SRS715491	1459	775	85	0.96	109	14
	MMRF10	Reproductive	Maize	CGATAT	SRS715492	831	532	55	0.96	78	10

Raw data were deposited under the study accession numbers SRP047229 for archaeal sequences in the NCBI Sequence Read Archive (SRA). Sample from one field (A–C) was randomly chosen and analyzed as representative of the field.

* number of sequences after quality analysis.

Partial 16S rRNA primers: Archaea: Arch344F (5′-ACGGGGYGCAGCAGGCGCGA), Arch934br (5′-GTGCTCCCCCGCCAATTCCT).

Adaptor primes: forward (5′-GATGGCCATTACGGCC), reverse (5′-GGTGGCCGAGGCGGCC).

Comparison of unplanted, maize and rice cultivated fields showed again a uniform distribution of the OTUs (Figure 5F). However, the OTUs assigned as Misc. Crenarchaeotic Group and unclassified were more abundant in the planted fields (rice, maize), while the unclassified OTU was more associated with the maize field. Similar to T-RFLP analysis an increase of *Methanosarcinaceae* in the maize fields and unplanted fields was observed, but not statistically significant (Supplement Figure 3A). In fact, no significant changes in the archaeal community composition were observed (ADONIS, *P* > 0.05).

**Figure 7:**
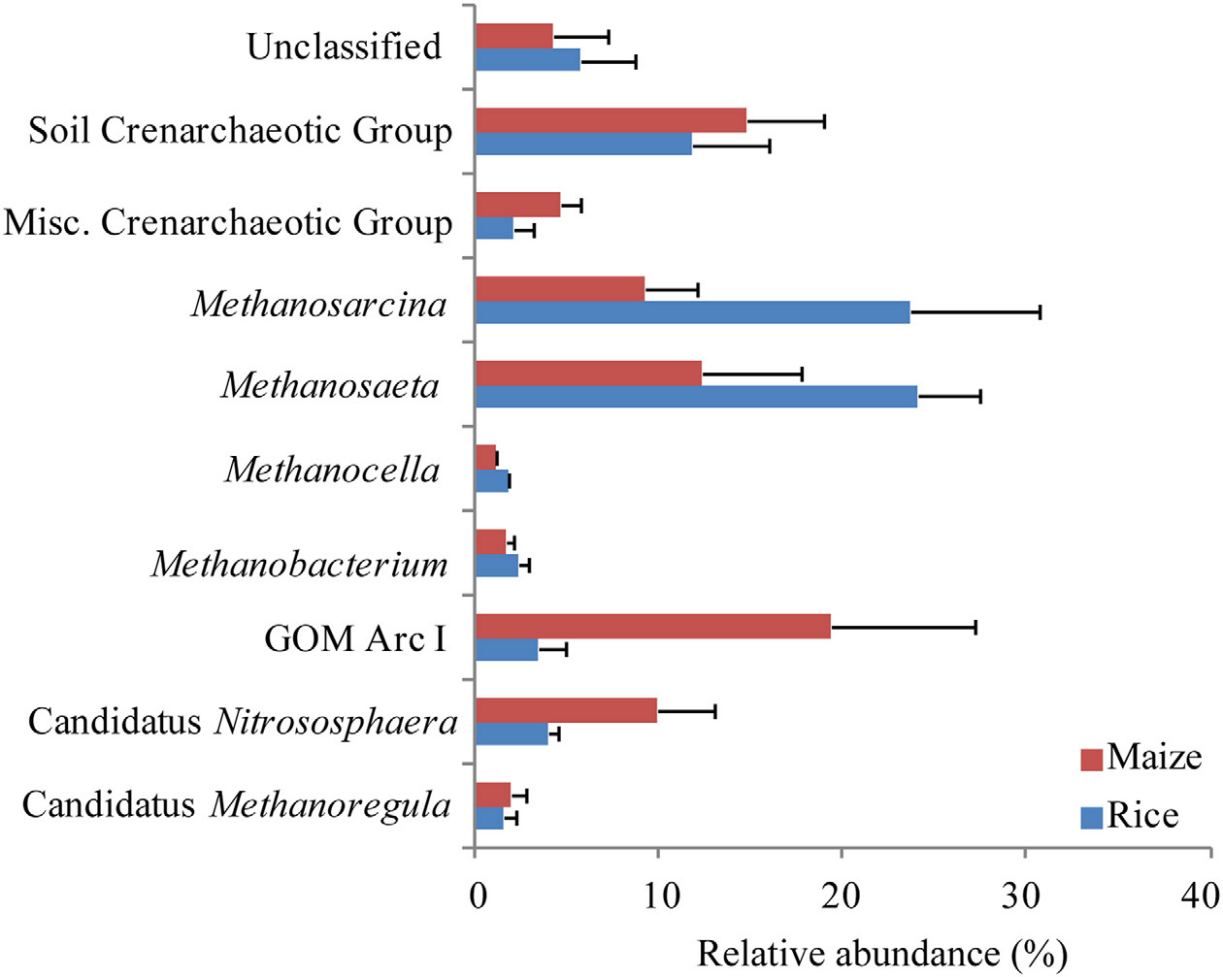
**Relative abundance of the archaeal OTUs in rice field soil**. Based on relative abundance top 30 OTUs derived from pyrosequencing of the archaeal 16S rRNA were grouped according to their phylogenetic assignment. OTUs were monitored in rice (blue) and maize (red) fields. Columns represent mean and bars standard errors of *n* = 3.

The sequences of ribosomal RNA were composed of the same archaeal lineages as the ribosomal gene-based community, but exhibited different relative abundances (Supplement Figure 3B). The most dominant class was *Methanomicrobia* (38–61% relative abundance) followed by Soil Crenarchaeotic Group (18–31%), Misc. Crenarchaeotic Group (4%), and *Methanobacteria* (2–6%). The archaeal community composition was not significantly different between fields cultivated with rice and maize on class, order and family level (ADONIS, *P* > 0.005). Nevertheless, specific archaeal groups changed in abundance. A significant increase of Soil Crenarchaeotic Group in the maize fields was observed (Supplement Figure 3B). Within Soil Crenarchaeotic Group Candidatus *Nitrososphaera* was higher in maize cultivated fields (data not shown). Only the top 30 OTUs representing up to 80% of all sequences were used for analysis (Figure 7). Again, there was a trend that methanogenic archaeal lineages (*Methanosarcina*, *Methanosaeta*, *Methanocella*, *Methanobacterium*) were more abundant in the rice than in the maize cultivated soil, and that non methanogenic groups (Soil Crenarchaeotic Group, GOM Arc I, Misc. Crenarchaeotic Group, Candidatus *Nitrososphaera*) were in particular observed in the maize field, but the trend was not statistically significant.

## Discussion

### Bacterial and archaeal communities at different rice growth stages

Total bacterial and archaeal 16S rDNA copy numbers increased during reproductive growth stage indicating growth. Recently, Lee et al. (2014) likewise described in a Korean rice field an increase in bacterial and archaeal copy numbers during rice plant growth followed by a decrease at plant maturity. Although the microbial numbers in our Philippine soil were one order of magnitude higher than in the Korean soil (Lee et al., 2014), in both soils the microbial numbers changed by only a factor of two over the season. A similar observation was made by Itoh et al. (2013) showing a moderate increase of the resident microbes during rice plant growth under flooded conditions in a Japanese rice field. Textbook knowledge tells that plants secrete a complex mixture of organic and inorganic compounds (rhizodeposits) via their root system. Several studies showed an increase in root exudation with rice plant growth reaching a maximum at reproductive stage and decreasing again toward maturity of the rice plants (Aulakh et al., 2001; Lu et al., 2002; Watanabe et al., 2004; Pump and Conrad, 2014). Therefore, it is likely that the modest increase from vegetative to reproductive and the decrease toward maturity of the bacterial and archaeal abundance is driven by root exudation.

In contrast to the change in the resident (16S rDNA) populations, the numbers of the active populations (16S rRNA) were stable and did not show seasonal dynamics. Studies comprehensively covering the resident and active archaeal as well as bacterial communities in rice fields during plant growth are hardly available (Itoh et al., 2013). The detection of ribosomal RNA is equivalent to that of ribosomes, which are indicative for actively dividing or actively metabolizing microbial cells (Blazewicz et al., 2013). Therefore, changes in the 16S rRNA pool caused by growing cells can be superimposed by fluctuations in the amount of active but non-growing cells. Additionally, it was shown that different taxa can have different numbers of 16S rDNA copies (e.g., Sukenik et al., 2012). Interestingly, the standard errors of the abundance of both active bacterial and archaeal populations were higher in the vegetative and reproductive plant growth phase and decreased with plant maturity. This may be an indication for the influence of root exudation affecting microbial activity. All together the data indicate that the bacterial and archaeal communities were composed of active and growing cells being enhanced during the reproductive growth stage possibly due to root exudation.

The composition of both the resident (16S rDNA) and the active (16S rRNA) bacterial community revealed only minor changes with the rice plant growth stages. Among the resident bacteria, only OTUs with negligible relative abundance were found to be specific for a particular plant growth stage. E.g., an OTU identified as *Anaeromyxobacter* was specific for the vegetative growth stage. *Anaeromyxobacter* spp. are known as iron reducers and are possibly important for carbon and iron dynamics in the rice rhizosphere (Ratering and Schnell, 2001; Treude et al., 2003). In rice fields oxidants like ferric iron are rapidly reduced (Conrad et al., 2014), but may be regenerated when plants allow O_2_ release from their roots, thus as during vegetative plant growth (Liesack et al., 2000). Among the active bacteria, *Cyanobacteria* were highest during the early vegetative growth phase possibly caused by the previous field preparation (i.e., puddling) mixing sun-exposed soil parts into the bulk. The increase in relative abundance of active *Verrucomicrobia* and *Bacteroidia* during reproductive plant growth may be a consequence of their ecophysiology, which is playing a role in carbon degradation (Sugano et al., 2005; Tanahashi et al., 2005; Kikuchi et al., 2007; Rui et al., 2009). A member of *Verrucomicrobia*, i.e., *Opitutus terrae*, was isolated from a paddy rice field as potential polysaccharolytic and saccharolytic and capable of hydrogen production (Chin et al., 1999, 2001). *Bacteroidia* are known key players in decomposition of rice plant residue (Weber et al., 2001; Akasaka et al., 2003) and *Bacteroidetes* prominent heterotrophs in rice field soil including a propionate-producing fermentative representative (Akasaka et al., 2003). The OTU based analysis showed that the methanotrophic *Methylocystis* became prominent before plant maturity. *Methylocystis*, a type-II methanotroph, has commonly been found in rice field soil (Murase and Frenzel, 2007; Shrestha et al., 2010). Methanotrophs are dependent on their primary substrate methane and oxygen. Oxygen was probably released by the roots during the reproductive growth phase of the rice plant. For example Gilbert and Frenzel (1998) reported radial oxygen loss by roots of up to 6 weeks old rice plants.

The T-RFLP analysis in the Philippine rice fields showed a relatively constant composition of the archaeal community over the season. Similar results had been obtained in an Italian rice field (Krüger et al., 2005). Our pyrosequencing data indicated an increase in relative abundance of the dominating methanogens (*Methanosarcinaceae, Methanosaetaceae*, *Methanobacteriaceae*, and *Methanocellaceae*) during reproductive growth stage, but this increase was statistically not significant. Within the order of *Methanosarcinales* GOM Arc I species were notably detected under all tested conditions and decreased during reproductive stage. GOM Arc I was formerly known as ANME-2d caused by phylogenetic relation to the anaerobic methanotrophs ANME-2 (Mills et al., 2005; Martinez et al., 2006). Nevertheless, the role of GOM Arc I in the methane biogeochemistry is still unclear (Lloyd et al., 2006; Knittel and Boetius, 2009). We speculate that the importance of these organisms which were previously detected in relatively high numbers in South Korean rice field soil (Ahn et al., 2014) has been underestimated and strengthen the need to identify their function in methane cycling.

All together rhizodeposition and oxygen release seemed to increase growth and activity of specific bacterial and archaeal lineages during the reproductive growth stage. However, changes over the season were only small and the resident and active microbial communities remained relatively conserved.

### Bacterial and archaeal communities in flooded and non-flooded fields

Rotation of the cultivated crop from paddy rice (flooded) to upland maize (non-flooded) changes the field conditions dramatically. In our study, we were dealing with flooded rice fields and non-flooded unplanted fields, which were then planted with maize, but kept under non-flooded conditions. Anaerobic degradation of organic matter to CH_4_ is only possible if the bulk of the soil is anoxic, such as in flooded fields. In non-flooded fields no or comparatively little anaerobic microbial activity is expected. Indeed, compared to the flooded fields CH_4_ emission was only minor from the non-flooded fields (Weller et al., 2014). Therefore, living conditions of obligately anaerobic microorganisms, such as methanogenic archaea and many fermenting bacteria were restricted.

The abundances of resident bacteria and archaea (16S rDNA) were lowest in unplanted fields, whereas they were highest in the flooded rice fields and intermediate in the non-flooded maize fields. The microbial populations apparently increased in number when the non-flooded fields were planted with maize, but did not reach the same level as in the flooded rice fields. Hence, microbial abundance was apparently affected by both flooding and the presence of vegetation. The low microbial abundance in unplanted fields was possibly due to the absence of release of organic material from roots and/or lack of fertilization, which allowed the microbes to grow to some extent in the maize fields. In these oxic soils, however, the number of microbes remained lower than in the anoxic flooded fields. Surprisingly, the abundance of ribosomal RNA, being indicative for active microbes, was in the same range for both unplanted and planted fields and for both non-flooded maize and flooded rice fields. Therefore, we assume that the microbial cells in non-flooded unplanted soil, and to some extend also the maize field soil, contained more ribosomal RNA than those in the flooded rice field soil. The rather high ratio of rRNA/rDNA in non-flooded fields was observed in most replicate samples, but there were a few replicates, which behaved differently. High ratios of rRNA/rDNA have also been observed in non-flooded Japanese rice fields but not been further discussed (Watanabe et al., 2007). However, numbers of rRNA decreasing with drainage have also been observed in a Japanese rice field (Itoh et al., 2013). At a first glance high ratios of rRNA/rDNA seem surprising, since anaerobic microorganisms should be less active in the unplanted and maize fields than in the flooded rice fields. However, it has been shown that even dormant cells harbor measureable amounts of 16S rRNA and that in some cases the 16S rRNA amount can even be significantly higher than in vegetative cells (Chambon et al., 1968; Sukenik et al., 2012). The maintenance of a high level of ribosomal RNA under unfavorable conditions is interpreted as preparedness for activity when conditions improve. Hence, we assume that numbers of anaerobic microorganisms decreased when the flooded rice fields were turned into non-flooded maize fields, but at the same time increased the cellular levels of rRNA (presumably ribosomes) as a stress response and possibly to be prepared for new flooding.

However, there may be additional explanations for the high ratio of rRNA/rDNA in the non-flooded soil. For example, during field preparation and after drainage soil structure gets disturbed, and this process may cause death of microbes by breaking up the cells. The nutrients of the dead cells become then available for the surviving microbes and may thus enhance their activity. The soil texture was a silt loam, which is characterized by a high water holding capacity. The high water holding capacity may have allowed the maintenance of anaerobic microniches with active populations of anaerobic microorganisms. Finally, drainage may have allowed an increase of the soil temperature, thus promoting the activity of the overall community which inhabits the anaerobic microniches.

Interestingly, the microbial community compositions were not much different between flooded and non-flooded fields. Although CCA analysis of the community based on T-RFLP revealed some differences in composition, the variance on the two CCA axes were less than 16%. Only the analysis by 454 pyrosequencing unveiled some changes in relative abundance of few bacterial groups but no dramatic community shifts. In the present study these bacterial lineages can be grouped due to their ecophysiology. *Spartobacteria* and *Sphingobacteria* were both described as aerobes and increased in their relative abundance in the non-flooded fields possibly due to decreasing water level and concomitant increased oxygen exposure. The first isolate of *Spartobacteria* was described as an aerobic heterotrophic bacterium able to grow on saccharide components of plant biomass (Sangwan et al., 2004). Additionally, some members of the *Sphingobacteria* were described as aerobes, while others are anaerobes or facultative anaerobes suggesting a dependence on oxygen levels (Janssen, 2006). The second group of bacterial lineages (*Bacteroidetes* and *Acidobacteria)* increasing in non-flooded fields is associated with their ability to sustain low substrate conditions and to degrade complex organic compounds under anaerobic conditions. For instance *Bacteroidetes* were frequently detected during rice plant residue decomposition (e.g., Weber et al., 2001; Rui et al., 2009) and have the ability to grow on various complex carbon substrates (Kirchman, 2002). Although *Acidobacteria* are widely distributed and highly abundant in soil environments little is known about their ecology (Lee and Cho, 2009). Various observations suggest that the chemo-organotrophic and oligotrophic *Acidobacteria* are adapted to low substrate availability highlighted by slow growth rates (e.g., Davis et al., 2005, 2011) and are able to decompose complex carbon compounds like xylan, cellulose and pectin (Eichorst et al., 2011). The last bacterial lineage more pronounced in the non-flooded fields was *Myxococcales*. Iron reducing *Anaeromyxobacter* are members of *Myxococcales* and represented the maturity of the order in the present study. During drainage regeneration of inorganic electron acceptors like ferric iron occurs. Therefore, it is likely that iron reducers out of *Myxococcales* are supported in their competition with methanogens for electron acceptors like hydrogen and acetate (Ratering and Conrad, 1998). In summary, the bacterial groups in the unplanted fields were characterized by their abilities to grow under oxic conditions and to degrade complex carbon substrates.

Similarly, the archaeal community composition was quite similar in non-flooded and flooded fields. This observation is consistent with previous studies showing that crop rotation including upland crop management affected archaeal communities only little (Watanabe et al., 2006, 2011; Fernandez Scavino et al., 2013). However, we observed a significant increase in relative abundance of *Methanosarcinaceae* in upland maize fields by T-RFLP analysis and a non-significant increase by pyrosequencing. In Japanese rice fields *Methanosarcinales* were a major group under both flooded and drainage conditions (Watanabe et al., 2009; Itoh et al., 2013). *Methanosarcina* spp. together with *Methanocella* spp. have also been found in dry ecosystems, such as upland soils and desert biological soil crusts (Nicol et al., 2003; Angel et al., 2012; Conrad et al., 2012; Aschenbach et al., 2013). These species possess a relatively large number of genes coding for oxygen-detoxifying enzymes (Erkel et al., 2006), thus probably allowing them to survive exposition to oxygen in dry soils (Angel et al., 2011, 2012). Therefore, it is likely that *Methanosarcina* spp. survived relatively well when the flooded rice fields were turned into non-flooded maize fields, thus increasing their relative abundance among the other archaea.

The Soil Crenarchaeotic Group also showed a relatively high abundance in the non-flooded maize fields. The ecophysiology of *Crenarchaeota* is largely unknown (Pester et al., 2011), although *Thaumarchaeota*, with potential for ammonia oxidation are found as a dominant archaeal group in aerated soils (e.g., Nicol et al., 2003). An upland pasture in Uruguay was reported to be dominated by *Crenarchaeota*/*Thaumarchaeota*, which decreased in relative abundance as soon as the soil was turned into a pasture-rice crop rotation (Fernandez Scavino et al., 2013). The predominance of *Methanosarcinaceae* and Soil Crenarchaeotic Group in non-flooded soils emphasize their capability to withstand temporal desiccation and oxygen stress.

### Conclusion

The bacterial and archaeal abundance and activity only moderately changed during rice growth most likely by the influence of rice plants and their root exudation. However, neither archaeal nor bacterial community composition changed much suggesting good adaptation to the conditions in the rice field. By contrast, the change from flooded rice to non-flooded cropping caused a comparatively stronger change in the microbial community composition, which however, was also not very dramatic. The relatively minor effect of change to non-flooded cropping was probably caused by the fact that the microbial communities in the rice field soil were historically adapted to regular drainage. This adaptation was also seen by the maintenance of a high ratio of ribosomal RNA per gene copy, being equivalent to a high number of ribosomes per cell, indicating a preparedness for change between unfavorable non-flooded to favorable flooded conditions for the methanogenic archaea and anaerobic bacteria resident in the rice field soil. The similarity in composition together with the statistically significant increase in ribosomal numbers imply that it was not so much specific members of the communities that regulated their ratios of rRNA/rDNA, but the communities in general that reacted upon the change from flooded to non-flooded state. We conclude that methods reducing greenhouse gas emission from rice fields like mid-season drainage and crop rotation (Wassmann et al., 2000; Li et al., 2006; Pittelkow et al., 2014) will have only little immediate effect on the bacterial and archaeal communities and thus, allow their function to be largely conserved over unfavorable periods.

### Conflict of interest statement

The authors declare that the research was conducted in the absence of any commercial or financial relationships that could be construed as a potential conflict of interest.
