# Supplementary material for: Seasonal dynamics of bacterial and archaeal methanogenic communities in flooded rice fields and effect of drainage
Source: Front Microbiol. 2015 Jan 8;5:752. doi: 10.3389/fmicb.2014.00752 (PMC4288041; doi:10.3389/fmicb.2014.00752)
Supplement: Supplementary file 1 [file DataSheet1.DOCX]

Supplement Table 1: Proportion of variance explained (percentage of total variation) by environmental variables determined by CCA for the resident (16s rDNA) and active (16S rRNA) bacterial and archaeal community based on T-RFLP.

| ***Community*** | ***Variable*** | ***% Variance explained*** | ***P-value*** |
| --- | --- | --- | --- |
| *16S rDNA Bacteria* | Field management  Growth stage  Gravimetric water content | 12.0  10.5  5.8 | 0.01*  0.01*  0.01* |
| *16S rRNA Bacteria* | Field management  Growth stage  Gravimetric water content | 14.4  21.4  5.7 | 0.01*  0.01*  0.01* |
| *16S rDNA Archaea* | Field management  Growth stage  Gravimetric water content | 16.2  22.8  7.5 | 0.01*  0.01*  0.02* |
| *16S rRNA Archaea* | Field management  Growth stage  Gravimetric water content | 15.7  11.2  11.7 | 0.01*  0.01*  0.01* |

*: significant. Significance was tested by ANOVA.


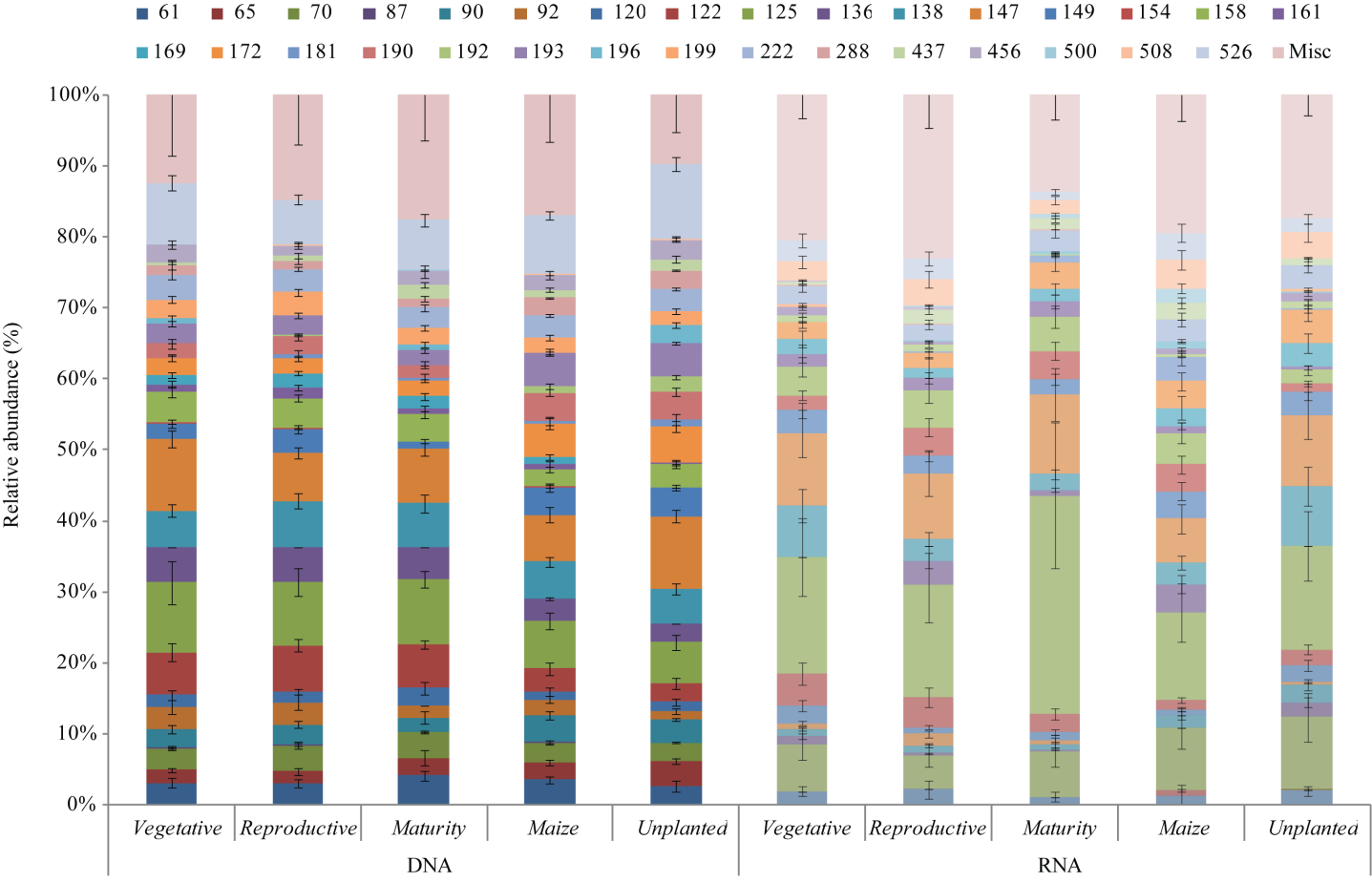


**Supplement Figure 1.** Histograms of the relative abundance of T-RFs obtained from T-RFLP analysis of bacterial 16S rDNA (left, dark columns) and rRNA (right, light columns) during rice plant growth and in non-flooded fields (unplanted, maize). Bacterial T-RFs with minimum 2% of relative abundance in at least one sample are mapped. Remaining T-RFs were summarized as Misc. Bars represent standard errors of n=9.

**
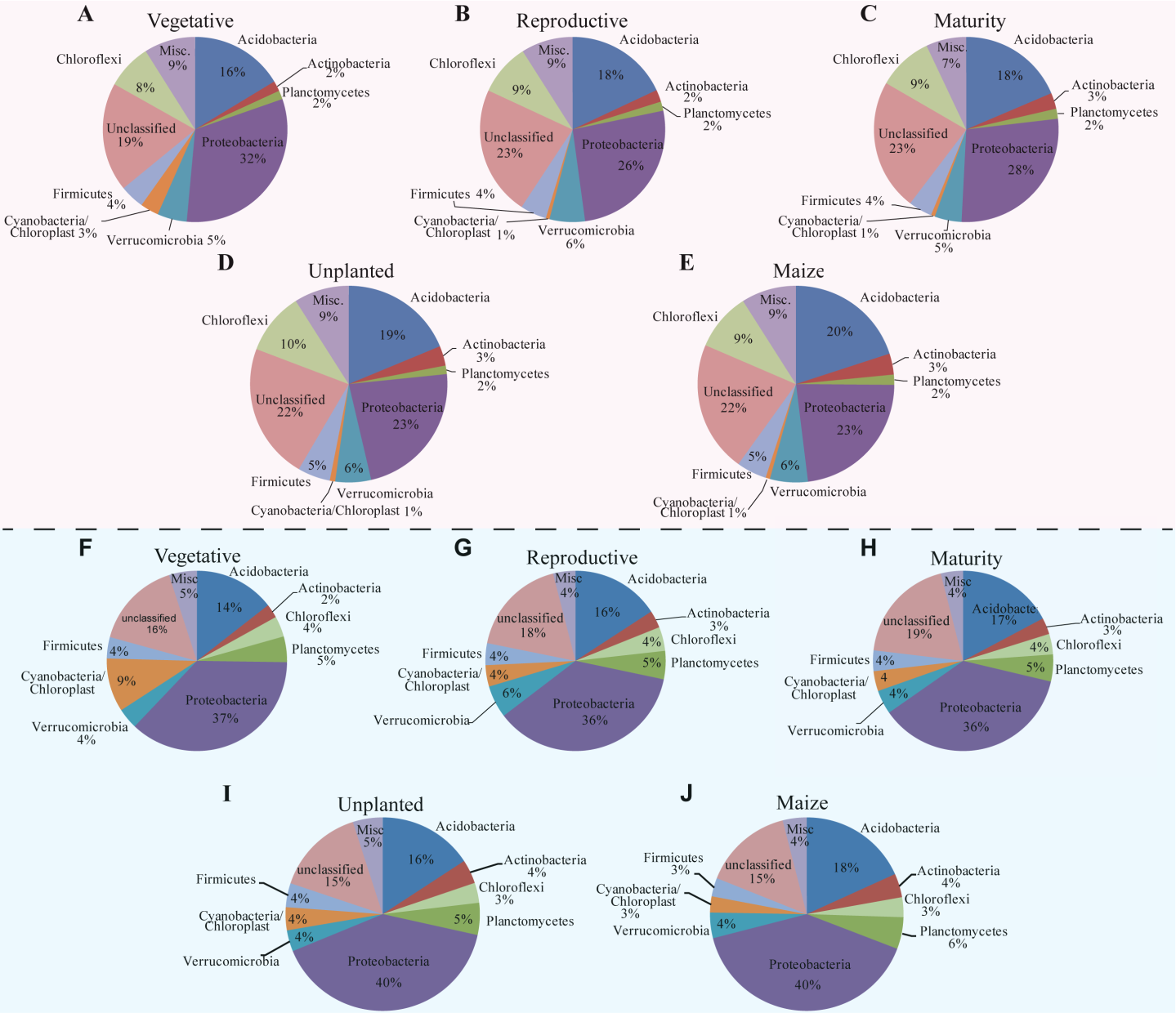
 Supplement Figure 2.** Relative abundance of the dominant bacterial phyla detected in rice field soil by pyrosequencing of 16S rDNA/rRNA. Bacterial phyla based on 16S rDNA during different rice plant growth stages **(A-C)** as well as in unplanted **(D)** and maize cultivated fields **(E)** and bacterial phyla based on 16S rRNA during different rice plant growth stages **(F-H)** as well as in unplanted **(I)** and maize cultivated fields **(J)** are displayed. Only phyla with a minimum of 2% relative abundance are shown, those with <2% are summarized as Misc. (n=3).


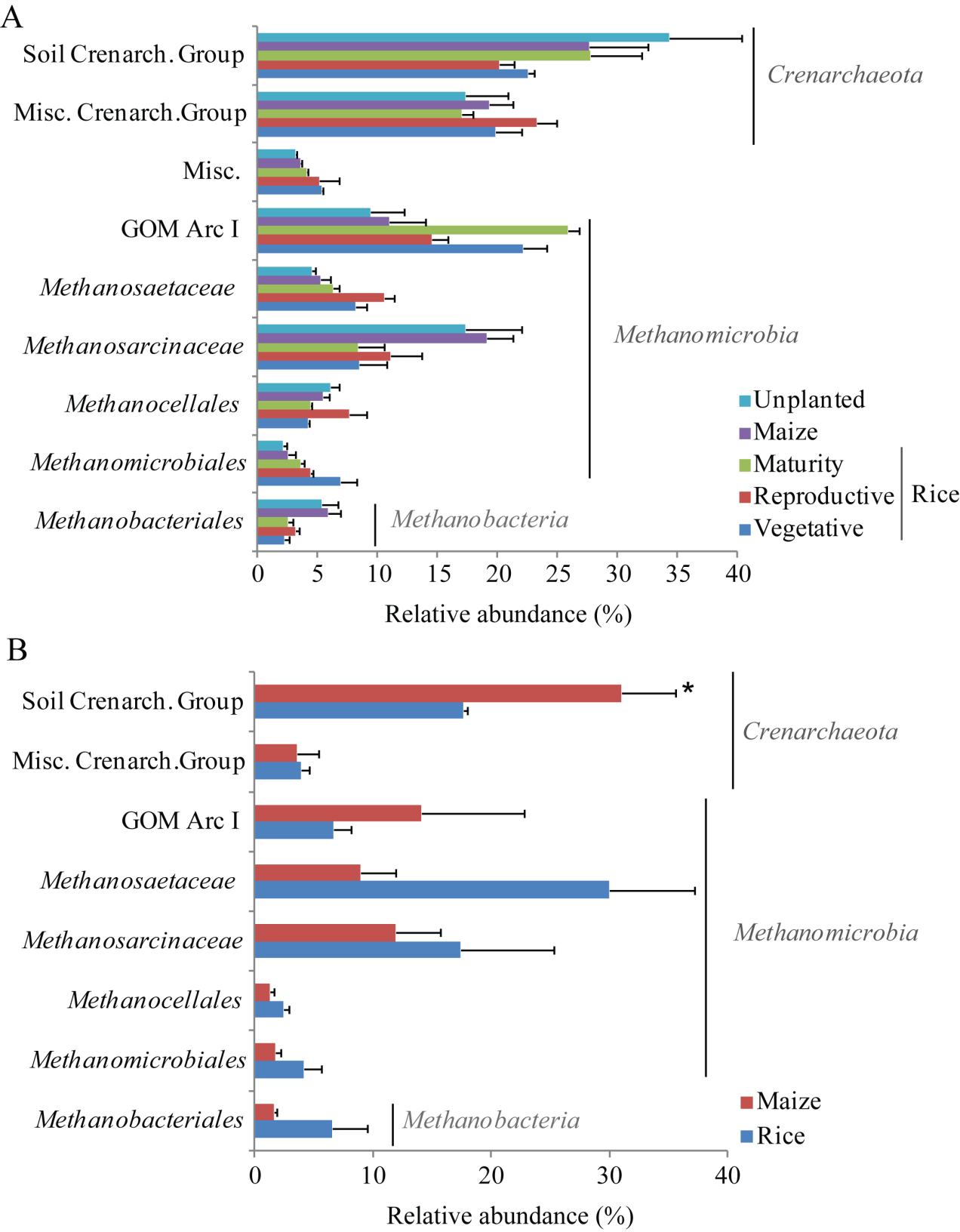
 **Supplement Figure 3.** Relative abundance of the archaeal lineages detected in rice field soil by pyrosequencing of 16S rDNA **(A)** and of 16S rRNA **(B)**. Archaeal lineages with minimum 2% of relative abundance in at least one sample are mapped. Remaining phyla were summarized as Misc. Columns represent mean and bars standard errors of n=3. Asterisk indicates significant difference.
